# A Height-Normalised Revised Drowning Index for Thoracic Fluid Burden: Associations with Water Type, Sex, Toxicology and Postmortem Interval

**DOI:** 10.3390/diagnostics16172870

**Published:** 2026-09-07

**Authors:** Eva Montanari, Cristian D’Ovidio, Tommaso D’Anna, Emina Dervišević, Claudia Rosa, Mariu Alexandru Apostol, Alberto Salomone, Enrico Gerace, Ismail Ozgur Can, Roberto Campagna, Angelo Montana

**Affiliations:** 1 Department of Excellence Biomedical Sciences and Public Health, Marche Polytechnic University, 60121 Ancona, Italy; 2 Section of Legal Medicine, Department of Medicine and Aging Sciences, G. d’Annunzio University of Chieti-Pescara, 66100 Chieti, Italy; cristian.dovidio@unich.it; 3 Department of Biomedicine and Prevention, Tor Vergata University of Rome, 00133 Rome, Italy; tommaso.danna@students.uniroma2.eu; 4 Tumor Immunology Unit, Department of Health Promotion, Mother and Child Care, Internal Medicine and Medical Specialties (PROMISE), University of Palermo, 90133 Palermo, Italy; 5 Legal Medicine Unit, University Hospital “Paolo Giaccone”, 90127 Palermo, Italy; 6 Department of Forensic Medicine, Faculty of Medicine, University of Sarajevo, 71 000 Sarajevo, Bosnia and Herzegovina; emina.dervisevic@mf.unsa.ba; 7 S.C. Medicina Legale, A.S.L. Città di Torino, 10135 Turin, Italy; claudia.rosa@aslcittaditorino.it; 8 Medicina Legale, A.S.L. TO4, Ivrea, 10015 Turin, Italy; marioapostol@hotmail.com; 9 Dipartimento di Chimica, Università degli Studi di Torino, 10125 Turin, Italy; alberto.salomone@unito.it; 10 Centro Regionale Antidoping e di Tossicologia “A. Bertinaria”, Orbassano, 10043 Turin, Italy; enrico.gerace@antidoping.piemonte.it; 11 Department of Forensic Medicine, School of Medicine, Dokuz Eylul University, 35340 Izmir, Türkiye; drforensicozgur@gmail.com; 12 Department for the Promotion of Human Science and Quality of Life, San Raffaele Roma University, Via di Val Cannuta, 247, 00166 Rome, Italy; roberto.campagna@uniroma5.it

**Keywords:** revised drowning index, thoracic fluid burden, lung weight, water type, toxicology, postmortem interval

## Abstract

**Background:** The forensic diagnosis of drowning lacks pathognomonic signs, and visceral weight-based indices normalised to spleen or heart weight have shown variable discriminant performance. We assessed a height-normalised revised Drowning Index (rDI) as a measure of thoracic fluid burden in confirmed drowning cases, not as a case–control test of drowning versus non-drowning deaths. **Methods:** Fifty-five institutional drowning cases (Ancona and Turin Province, Italy, 2008–2020) were analysed. The rDI and the original spleen-normalised Drowning Index (DI) were calculated. A multivariable linear regression assessed sex, water type, and toxicological status as simultaneous covariates; a receiver operating characteristic (ROC) analysis evaluated the within-cohort classification of water type; and the associations with the postmortem interval (PMI) were explored separately. **Results:** The rDI was independently associated with the male sex (standardised β = 0.391, *p* = 0.002) and saltwater drowning (β = 0.331, *p* = 0.011; adjusted R^2^ = 0.269). The three benzodiazepine-positive institutional cases had a lower rDI in the univariate analysis (*p* = 0.019), but the association was not retained in the multivariable model or in a freshwater-restricted sensitivity analysis. Alcohol exposure showed no association. The water-type ROC analysis yielded an area under the curve of 0.727 (95% CI 0.553–0.871); the distributions overlapped substantially. The original DI was not associated with the variables studied. The rDI showed no significant association with the PMI within the range represented by all but one case (1–12 days). **Conclusions:** The rDI quantified the variation in the thoracic fluid burden within this cohort of confirmed drownings and showed moderate, exploratory water-type classification. It is not a stand-alone diagnostic test, and its discrimination ability between drowning and non-drowning deaths was not assessed. Dedicated case–control and external validation studies are required before forensic cut-offs can be recommended.

## 1. Introduction

Drowning deaths remain a challenging diagnosis in forensic pathology because no single pathognomonic sign exists. The classical autopsy picture—hyperexpanded lungs with overlapping anterior margins, frothy fluid in the tracheobronchial airway, pleural effusion, and emphysema aquosum on the histology—is informative but inconstant [1]: a substantial minority of bodies recovered from water present with “dry lungs”, with reported frequencies ranging from 2% to 18% depending on the cut-off adopted, generally based on a combined lung weight below 1000 g [2]. This pattern, attributed to laryngospasm and reflex cardiac arrest triggered by the initial contact of the upper airway with liquid, is conceptually important because it can mimic, or be mistaken for, drowning without significant aspiration (“dry drowning”) [3].

Building on these diagnostic limitations, several authors have proposed normalised indices to make comparisons between drowning cases more objective. Nishitani et al. introduced the Drowning Index (DI), expressed as the ratio of combined lung weight and pleural effusion to spleen weight [4], while Zhu et al. proposed an analogous ratio using the heart weight as the denominator [5]. In an independent validation, Wardak et al. found that both indices lacked sufficient discriminant power to differentiate drowning from mechanical asphyxia or acute myocardial infarction [6]. Testing of the spleen-based DI in opioid and multidrug intoxication deaths likewise showed that high values are not specific to drowning [7]. More recently, Nagar et al. compared 75 freshwater drownings with 64 sudden cardiac deaths and 40 hanging deaths and reported useful, but incomplete, case–control discrimination by the original DI [8]. Collectively, these studies show why any diagnostic claim requires a defined non-drowning comparison group and external validation; organ weight indices should remain adjuncts to circumstantial, macroscopic, and histological evidence.

The marked male predominance among drowning fatalities is also relevant to the interpretation of thoracic measurements. In an Amsterdam cohort from 2011 to 2015, males accounted for 82% of fatal drowning cases [9]. This imbalance is usually attributed to differential exposure, including alcohol or drug use, risk-taking behaviour, and environmental circumstances. Whether sex-related differences in physique and lung size also influence postmortem lung weight and pleural fluid measurements requires explicit consideration.

Against this background, the present study proposes a height-normalised revised Drowning Index (rDI), using the same numerator—combined lung weight and pleural effusion—but replacing the spleen weight with body height. Throughout this article, the abbreviation DI is reserved for the original spleen-normalised index described by Nishitani et al. [4], whereas rDI denotes the height-normalised measure evaluated here. Height is a stable anthropometric parameter and is less susceptible than visceral organ weight to antemortem disease and postmortem change.

Accordingly, the present study aimed to evaluate whether the rDI is associated with water type, sex, and toxicological status in a 55-case series of confirmed drownings, using univariate and multivariable approaches. The secondary aims were to assess its exploratory within-drowning classification of water type and to examine its stability across the PMI encountered in routine forensic casework. Because no non-drowning comparison group was included, the study was not designed to estimate the ability of rDI to distinguish drowning from other causes of death.

## 2. Materials and Methods

### 2.1. Study Population

Drowning cases autopsied between 2008 and 2020 were retrospectively retrieved from the archives of the Sections of Legal Medicine serving Ancona, Marche, and Turin Provinces, Piedmont, Italy. Cases were included in the study database when complete anthropometric, circumstantial, autopsy, and toxicological data required for the planned analyses were available.

Cases were eligible when the body was recovered submersed, defined as the entire body being underwater, or immersed, defined as at least the face or airways being covered by water. Eligibility also required that no traumatic or violent cause of death was identified. The diagnosis of drowning was established from circumstantial information integrated with classical autopsy and histological findings, including hyperexpanded lungs, pinkish frothy fluid within the tracheobronchial tree, acute emphysema with rupture of interalveolar septa, and intra-alveolar eosinophilic fluid.

The PMI was the interval documented in the retrospective case file between the recorded or estimated time of death and the autopsy. The archive did not uniformly distinguish witnessed, precisely timed deaths from unwitnessed deaths estimated from last-known alive, recovery, or scene information; the recorded PMI therefore cannot be interpreted as an exact biological time of death in every case. Putrefactive findings were used to interpret markedly prolonged PMI cases qualitatively, but no standardised decomposition-derived time estimate was analysed. The PMI was recorded in whole days. The original inclusion criterion was 1–4 days; cases with longer recorded intervals were retained for exploratory assessment of the relationships among the PMI, lung weight, pleural effusion, and rDI, and were treated cautiously because of decomposition-related changes.

The toxicological data were reviewed for each institutional case. The screening results were classified as negative, positive for ethanol alone, or positive for benzodiazepines, with the latter group including cases in which benzodiazepines were detected alone or with other substances. Only the 55 cases from the three participating jurisdictions were included in the cohort and in all statistical analyses.

### 2.2. Autopsy and Toxicological Protocol

During each autopsy, right and left lung weights and right and left pleural effusion weights were recorded separately, together with height and other anthropometric parameters. Histological examination of haematoxylin–eosin-stained sections formed part of integrated cause-of-death assessment. Toxicological analyses were carried out on central or peripheral blood and, when available, urine. Preliminary screening for amphetamines, barbiturates, benzodiazepines, cannabinoids, methadone, cocaine, and opiates was performed by Enzyme-Multiplied Immunoassay Technique (EMIT, Abbott Laboratories, Abbott Park, IL, USA); ethanol was quantified by headspace GC-MS; benzodiazepines and other pharmaceutical drugs were confirmed and quantified by UHPLC-MS/MS.

The revised Drowning Index (rDI) was calculated for every institutional case as

rDI (g/cm) = (right lung weight + left lung weight + right pleural effusion weight + left pleural effusion weight)/height (cm)

### 2.3. Statistical Analysis

The continuous variables are reported as the mean ± SD or, for markedly skewed PMI values, as the median and interquartile range. The between-group comparisons used two-sided Student’s *t*-tests (or Welch’s *t*-tests for unequal variances), with Mann–Whitney U tests as non-parametric checks. A multivariable linear regression was used to assess sex, water type, benzodiazepine positivity, and alcohol positivity as simultaneous covariates of rDI. The ROC analysis evaluated the exploratory within-cohort classification of saltwater versus freshwater drowning; the operating point with the largest Youden index was reported together with two descriptive thresholds. This ROC analysis did not evaluate drowning versus non-drowning deaths, and the thresholds were not externally validated. A freshwater-restricted sensitivity analysis adjusted for sex was used to examine the benzodiazepine association. Associations of PMI with lung weight, pleural effusion, the rDI numerator, and rDI were examined using correlation and water-type-adjusted linear models; cases with markedly prolonged PMI were examined separately. Statistical significance was set at *p* < 0.05. Analyses were performed using IBM SPSS Statistics, version 29.0 (IBM Corp., Armonk, NY, USA).

## 3. Results

### 3.1. Cohort Characteristics

The institutional cohort comprised 55 drowning cases, including 38 males and 17 females. Their ages ranged from 16 to 85 years, with a mean of 55.6 ± 20.1 years, while their height ranged from 144 to 190 cm, with a mean of 168.2 ± 11.1 cm. Forty-one cases occurred in saltwater and 14 in freshwater. The postmortem interval ranged from 1 to 70 days, with a median of 2 days and an interquartile range of 2–4 days.

### 3.2. Toxicological Findings

The toxicological screening was positive in 16 of the 55 cases (29.1%): 13 for ethanol alone and three for benzodiazepines (bromazepam in one case; lormetazepam/lorazepam in two cases); the remaining 39 cases (70.9%) were drug-free. No case was positive for both ethanol and benzodiazepines.

### 3.3. Factors Associated with the Revised Drowning Index

The rDI was higher in saltwater than in freshwater drownings (10.99 ± 2.97 vs. 7.91 ± 3.58 g/cm; Welch’s *t*-test *p* = 0.009; Mann–Whitney *p* = 0.012) and in males than in females (11.19 ± 2.90 vs. 8.02 ± 3.44 g/cm; *p* = 0.003; Mann–Whitney *p* = 0.002). No difference emerged for alcohol exposure (10.48 ± 1.85 g/cm in positive cases, n = 13, vs. 10.37 ± 3.64 g/cm in negative cases, n = 42; *p* = 0.893). The benzodiazepine-positive cases had a lower rDI (6.55 ± 1.40 g/cm, n = 3) compared with benzodiazepine-negative cases (10.42 ± 3.35 g/cm, n = 52; *p* = 0.019; Mann–Whitney *p* = 0.040). This small univariate comparison should be interpreted cautiously. No significant difference in rDI emerged between cases with a PMI ≤ 1 day and those with a PMI > 1 day (11.42 ± 3.44 vs. 9.91 ± 3.34 g/cm; *p* = 0.208), nor for the original DI (15.31 ± 6.10 vs. 15.40 ± 8.58; *p* = 0.971).

### 3.4. Multivariable Analysis

To address possible confounding among sex, water type, and drug exposure, a multivariable linear regression model including sex, water type, benzodiazepine positivity, and alcohol positivity as simultaneous covariates was fitted (n = 53). The male sex (standardised β = 0.391, *p* = 0.002) and saltwater drowning (β = 0.331, *p* = 0.011) remained independently associated with a higher rDI, whereas neither benzodiazepine positivity (β = −0.137, *p* = 0.282) nor alcohol positivity (β = −0.016, *p* = 0.897) was significant after adjustment (adjusted R^2^ = 0.269) (Table 1). In a freshwater-restricted sensitivity analysis adjusted for sex, the benzodiazepine association was not significant (rDI 6.30 ± 1.88 g/cm, n = 2, vs. 8.18 ± 3.78 g/cm, n = 12; adjusted *p* = 0.63). Thus, the univariate signal may partly reflect the unequal distribution of the three benzodiazepine-positive cases across water types rather than an isolated drug effect.

### 3.5. Exploratory Water-Type Classification with the rDI

The ROC analysis showed moderate within-cohort classification of water type by the rDI, with an area under the curve of 0.727 (95% CI 0.553–0.871). The threshold with the greatest Youden index was an rDI ≥ 9.0 g/cm (sensitivity 73%; specificity of 64% for saltwater). An rDI ≥ 12.5 g/cm had 100% specificity but 32% sensitivity, while all saltwater cases had an rDI ≥ 5.4 g/cm (sensitivity 100%; specificity 36%) (Table 2). The substantial overlap between freshwater and saltwater distributions (approximately 5.4–12.5 g/cm) precluded stand-alone classification. These internally derived operating points are descriptive and require external validation.

### 3.6. Comparison with the Original Drowning Index

The original DI [(combined lung weight + pleural effusion weight)/spleen weight] was calculated in 53 of 55 cases for which the spleen weight was available. In contrast with the rDI, the original DI was not significantly associated with water type (saltwater 16.02 ± 8.50 vs. freshwater 13.42 ± 6.51; *p* = 0.260), sex (male 15.63 ± 6.61 vs. female 14.79 ± 10.99; *p* = 0.779), alcohol exposure (positive 13.42 ± 5.46 vs. negative 15.95 ± 8.67; *p* = 0.232), benzodiazepine exposure (positive 10.65 ± 5.07 vs. negative 15.66 ± 8.17; *p* = 0.221), or PMI category (≤1 day 15.31 ± 6.10 vs. >1 day 15.40 ± 8.58; *p* = 0.971). These within-drowning comparisons did not test the original DI’s intended discrimination between drowning and non-drowning deaths.

### 3.7. Effect of Postmortem Interval

The PMI ranged from 1 to 70 days (median 2; IQR 2–4); only two cases exceeded 10 days (12 and 70 days). The 70-day case was treated as an extreme decomposition-related outlier, whereas the 12-day case remained in the 1–12-day analysis but was interpreted cautiously. Within 1–12 days (n = 54), the total lung weight showed a weak inverse correlation with the PMI (r = −0.27, *p* = 0.047; −47 g/day after adjustment for water type, *p* = 0.048), whereas pleural effusion showed no association (r = −0.04, *p* = 0.785; adjusted *p* = 0.84). Neither the rDI numerator nor rDI showed a significant association with the PMI after exclusion of the 70-day case (*p* = 0.15 and *p* = 0.16, respectively; Spearman *p* = 0.35–0.50 in the full sample). Because the retrospective PMIs were not exact in every unwitnessed case and were recorded in whole days, these analyses are exploratory and should not be interpreted as proving temporal stability.

## 4. Discussion

The relationship between the postmortem interval and fluid redistribution between lung parenchyma and the pleural cavity has been investigated in several larger drowning series. Zhu et al. [10] described a gradual postmortem decrease in lung weight accompanied by a reciprocal increase in pleural effusion, consistent with progressive transudation from lung to pleural space. In a cohort of 130 drowning cases, Ishigami et al. likewise reported decreasing lung weight and increasing pleural effusion with a longer PMI, most pronounced beyond three days [11].

The use of combined lung weight and pleural effusion as the numerator is supported by morphometric evidence that an isolated lung weight may decrease with an increasing PMI, while pleural effusion may increase through postmortem transudation. The combined thoracic fluid burden may therefore capture the drowning-related pulmonary fluid load more consistently than lung weight alone. This is the rationale for the numerator of rDI.

In our series, lung weight showed a weak inverse correlation with PMI, whereas pleural effusion did not increase in parallel and the rDI was not significantly associated with the PMI. This partly overlaps with the lung weight trend reported by Zhu et al. [10], but not with their reciprocal increase in pleural effusion.

By contrast, other authors have not found correlations between lung weight or pleural effusion and PMI [4,5,12].

This apparent discrepancy may reflect limited statistical power at the extreme end of the PMI/immersion-interval distribution. The cohorts analysed by Zhu et al. and Ishigami et al. included approximately 70–130 cases, with many bodies recovered after several days in water [10,11]; by contrast, only two cases in our series had a PMI longer than 10 days. The 70-day case showed a combined lung weight of 420 g and no pleural effusion and accounted for much of the apparent late decline. Yorulmaz et al. reported that pleural fluid accumulation may decrease with advanced decomposition, potentially because fluid diffuses or leaks from the thoracic cavity [13]. The 12-day case in our series remained within the range observed at shorter PMIs. Larger series with more long-PMI cases and better-resolved time estimates are needed to define any non-linear late changes.

Within the early-to-intermediate PMI range represented by most cases, the combined numerator and rDI showed no statistically detectable trend. This does not establish a mechanism of continuous fluid redistribution or prove PMI invariance: the data are also compatible with limited power, imprecise time estimates, and a later non-linear decline after advanced decomposition. The height was used as an anthropometric denominator, not as a biological marker of PMI. The rDI should therefore be interpreted as an exploratory morphometric measure whose temporal robustness requires prospective validation.

Saltwater drowning and male sex were independently associated with a higher rDI in the multivariable model (adjusted R^2^ = 0.269).

The higher rDI observed in saltwater drowning is consistent with the osmotic mechanism by which hypertonic seawater draws fluid across the alveolar–capillary membrane, increasing the pulmonary fluid burden [5,10,11]. The sex association should not, however, be attributed to a drowning-specific alveolar mechanism on the basis of these data. The standing height alone does not fully capture the sex-related differences in thoracic dimensions or lung volume; contemporary spirometric reference equations retain sex as a predictor in addition to height [14]. Moreover, postmortem lung and pleural fluid weights are not equivalent to the premortem lung capacity. Residual differences in physique may therefore contribute to the observed association. Future studies should collect body weight, body mass index, thoracic circumference, and predicted lung volume and should examine sex-specific or multivariable anthropometric normalisation.

The three benzodiazepine-positive institutional cases showed the lowest rDI in the univariate analysis.

This small subgroup does not support a causal or drug-specific inference. The association was not retained after multivariable adjustment or freshwater restriction and may reflect water type, sex, co-exposures, or chance. Effects of sedation on consciousness, airway protective reflexes, and aspiration remain possible mechanistic hypotheses, but they cannot be distinguished in the present retrospective dataset. The potential forensic toxicological relevance of lorazepam and lormetazepam is supported by their established central nervous system depressant effects, which may result in marked sedation or stupor and could theoretically increase vulnerability to respiratory compromise or prolonged laryngospasm [15]. However, the present data do not establish such a mechanism or demonstrate a specific contribution of either drug to the observed association. Accordingly, the benzodiazepine finding should be regarded solely as hypothesis-generating.

Alcohol exposure showed no association with the rDI in either the univariate or multivariable analysis. Lunetta et al. found no significant association between blood alcohol concentration and low combined lung/pleural liquid weight in a large drowning series [2]. By contrast, Poulain et al. reported a greater absolute lung weight in cases with a positive blood alcohol concentration or a higher toxicological participation score, possibly reflecting intoxication-related pulmonary oedema or congestion superimposed on drowning-related oedema [16]. Poulain et al. analysed the absolute lung weight without the anthropometric normalisation used here; no corresponding association was detected for the rDI in our cohort.

The comparison between indices in the present study is limited to the variation within confirmed drowning cases. In this setting, the rDI was associated with water type and sex, whereas the original spleen-normalised DI was not associated with the variables examined. This finding does not establish that the rDI is superior for distinguishing drowning from non-drowning deaths, because no non-drowning control group was available.

The narrower variability in the rDI may partly reflect its denominator. Spleen and heart weights are influenced by disease and decomposition, whereas height is fixed at death. However, height does not capture all differences in body build or lung capacity, and the observed coefficients of variation (52% for the original DI and 27% for the rDI) cannot by themselves demonstrate diagnostic superiority. Future models should compare several anthropometric normalisation strategies in external datasets.

The practical utility of rDI is deliberately limited. The water type is usually known from the recovery circumstances, and the rDI adds little when those circumstances are unequivocal. Its possible role is as a quantitative consistency check when the scene information is incomplete or contested, or as a standardised research measure of thoracic fluid burden. The moderate AUC and substantial freshwater–saltwater overlap mean that the rDI cannot determine the cause of death; reconstruct the immersion environment on its own; or replace circumstantial, autopsy, histological, and toxicological assessments. The reported operating points are internally derived and should not be applied as validated forensic cut-offs.

This study has several limitations. Foremost, it includes confirmed drowning cases only and has no non-drowning comparison group; it therefore cannot assess the core diagnostic question of discriminating drowning from mechanical asphyxia, sudden cardiac death, intoxication, or other deaths in bodies recovered from water. The recent comparative study by Nagar et al. illustrates the required case–control framework for evaluating the original DI [8]. Validation of rDI will similarly require prospectively defined comparison groups, external cohorts, and prespecified thresholds. The small benzodiazepine-positive subgroup (n = 3) precludes causal inference. The retrospective, multicentre design may also permit centre-related differences in case mix or measurement practice, and the available data do not support adjustment for physique beyond height. The PMI was recorded in whole days and was not uniformly distinguishable as an exact witnessed time versus a case file estimate in unwitnessed deaths; putrefaction was not represented by a standardised quantitative score. Only two cases had a PMI > 10 days, limiting assessment of advanced decomposition. Diatom and strontium testing were not performed; case inclusion relied on integrated circumstantial, macroscopic, and histological findings. These limitations make all water-type operating points and temporal findings exploratory.

## 5. Conclusions

In this series of 55 confirmed drownings, the height-normalised rDI was associated with water type and sex and was not significantly associated with the recorded PMI across 1–12 days, whereas the original spleen-normalised DI was not associated with the variables examined. The lower rDI in three benzodiazepine-positive cases was not independently supported after adjustment. The exploratory water-type classification was moderate and the distributions overlapped substantially; the rDI is therefore a descriptive adjunct rather than a stand-alone test. Because no non-drowning comparison group was included, the study does not establish diagnostic discrimination for drowning. Dedicated case–control and external validation studies, following comparison group approaches such as that of Nagar et al. [8], and including mechanical asphyxia, sudden cardiac death, and intoxication, are required before the rDI can be considered for diagnostic use.

## Figures and Tables

**Table 1:** Multivariable linear regression model for the revised Drowning Index (rDI).

Predictor	Unstandardised β	SE	95% CI	Standardised β	*p*
Male sex	2.761	0.865	1.023 to 4.500	0.391	0.002
Saltwater drowning	2.493	0.945	0.593 to 4.392	0.331	0.011
Benzodiazepine positivity	−1.926	1.771	−5.486 to 1.635	−0.137	0.282
Alcohol positivity	−0.122	0.938	−2.007 to 1.763	−0.016	0.897

The rDI is entered as the dependent variable. Sex, water type, benzodiazepine positivity, and alcohol positivity are included as simultaneous covariates. Model n = 53; R^2^ = 0.325; adjusted R^2^ = 0.269. β: regression coefficient; SE: standard error; CI: confidence interval.

**Table 2:** Internally derived operating points for exploratory saltwater-versus-freshwater classification by rDI. AUC = 0.727 (95% CI 0.553–0.871).

rDI Threshold (g/cm)	Sensitivity for Saltwater	Specificity for Saltwater	Interpretation
≥9.0	73%	64%	Largest Youden index
≥12.5	32%	100%	High specificity; low sensitivity
≥5.4	100%	36%	All saltwater cases at or above threshold

## Data Availability

The data presented in this study are available on request from the corresponding author. The data are not publicly available because they are derived from autopsy records subject to confidentiality restrictions.

## References

[1] Piette M.H.A., De Letter E.A. (2006). Drowning: Still a difficult autopsy diagnosis. Forensic Sci. Int..

[2] Lunetta P., Modell J.H., Sajantila A. (2004). What is the incidence and significance of “dry-lungs” in bodies found in water?. Am. J. Forensic Med. Pathol..

[3] Armstrong E.J., Erskine K.L. (2018). Investigation of Drowning Deaths: A Practical Review. Acad. Forensic Pathol..

[4] Jørgensen K.B., Busch J.R., Wingren C.J. (2026). Diagnostic utility of the drowning index: A review of the literature and analysis of 7105 Danish autopsy cases. Forensic Sci. Int..

[5] Zhu B.L., Quan L., Ishida K., Oritani S., Li D.R., Taniguchi M., Kamikodai Y., Tsuda K., Fujita M.Q., Nishi K. (2003). Lung–heart weight ratio as a possible index of cardiopulmonary pathophysiology in drowning. Leg. Med..

[6] Wardak K.S., Buchsbaum R.M., Walyzada F. (2014). The Drowning Index: Implementation in drowning, mechanical asphyxia, and acute myocardial infarct cases. J. Forensic Sci..

[7] Ellis L.T., Opsahl M., Duff D.J., Stacy C.C. (2019). Application of the Drowning Index to opioid and multidrug intoxication deaths: A retrospective analysis. Acad. Forensic Pathol..

[8] Nagar N., Bastia B.K., Singi Y., Dabhi D., Nagar K. (2025). Assessment of the drowning index in diagnosing freshwater drowning: A comparative study. J. Forensic Sci..

[9] Reijnen G., van de Westeringh M., Buster M.C., Vos P.J.E., Reijnders U.L.J. (2018). Epidemiological aspects of drowning and non-fatal drowning in the waters of Amsterdam. J. Forensic Leg. Med..

[10] Zhu B.L., Quan L., Li D.R., Taniguchi M., Kamikodai Y., Tsuda K., Fujita M.Q., Nishi K., Tsuji T., Maeda H. (2003). Postmortem lung weight in drownings: A comparison with acute asphyxiation and cardiac death. Leg. Med..

[11] Ishigami A., Kashiwagi M., Ishida Y., Hara K., Nosaka M., Matsusue A., Yamamoto H., Waters B., Kondo T., Kubo S. (2021). A comparative study of pleural effusion in water area, water temperature and postmortem interval in forensic autopsy cases of drowning. Sci. Rep..

[12] Sugimura T., Kashiwagi M., Matsusue A., Hara K., Kageura M., Kubo S. (2010). Application of the drowning index to actual drowning cases. Leg. Med..

[13] Yorulmaz C., Arican N., Afacan I., Dokgoz H., Asirdizer M. (2003). Pleural effusion in bodies recovered from water. Forensic Sci. Int..

[14] Quanjer P.H., Stanojevic S., Cole T.J., Baur X., Hall G.L., Culver B.H., Enright P.L., Hankinson J.L., Ip M.S.M., Zheng J. (2012). Multi-ethnic reference values for spirometry for the 3–95-yr age range: The global lung function 2012 equations. Eur. Respir. J..

[15] Montanari E., Siliquini S., Lo Faro A.F., Rotolo M.C., Busardò F.P., Vidua R.K., De Simone S., Montana A. (2025). Benzodiazepines induced stupor in a four-year-old child: When EEG meets toxicology. Egypt. J. Forensic Sci..

[16] Poulain C., Mathieu O., Gouetta V., Baccino É., Peyron P.A. (2023). Influence of the toxicological status on the diagnosis of fatal drowning. Int. J. Leg. Med..

